# Design and Implementation of a Novel IoT Architecture for Data Release System Between Multiple Platforms: Case of Smart Offshores

**DOI:** 10.3390/s25113384

**Published:** 2025-05-28

**Authors:** Bernard Marie Tabi Fouda, Lei Wang, Dezhi Han, Paul Claude Ngoumou, Jacques Atangana

**Affiliations:** 1 Department of Internet of Things Engineering, College of Information Technology, Shanghai Jianqiao University, Shanghai 201306, China; 2 Department of Applied Physics, Faculty of Science, University of Ebolowa, Ebolowa 118, Cameroon; ngpclaude@yahoo.fr (P.C.N.); atanganajaques@yahoo.fr (J.A.); 3 Department of Computer Science, College of Information Engineering, Shanghai Maritime University, Shanghai 201306, China; dzhan@shmtu.edu.cn

**Keywords:** IoT architecture, data release, dynamic configuration, IEC60870-5-104 protocol, smart offshores, photoelectric composite submarine cables

## Abstract

The evolution of automation has reached marine operations in general and offshore operations in particular. Many facilities in these areas use the Internet of Things (IoT) to consolidate processes and improve data release systems. In addition, the IEC60870-5-104 protocol (IEC104) enables remote data release. This paper introduces and develops a novel IoT architecture that enables the continuous acquisition, evaluation, and release of data between platforms. Continuous data release is based on a dynamic configuration (DC) approach using the IEC104 protocol (DC-IEC104). The proposed approach thoroughly analyzes the structural model and communication process and then proposes a set of design tables according to the information object (type and amount) of the data to be released. In the application case, the data of the photoelectric composite submarine cables were successfully released with an average mean square error of 3.78 and an average processing time of 1.083 s. These results have been proven to be better compared to those obtained using three other approaches for data release.

## 1. Introduction

### 1.1. Study Overview and Challenges

Offshore operations and submarine cables are no longer exceptions to the automation revolution. An increasing number of maritime fields include a certain type of automation used to improve the performance of their systems and their quality of service. With the Internet of Things (IoT) and the arrival of mobile networks (5G), it is possible to connect multiple devices with high bandwidth and low latency [1,2]. In addition, DC using IEC enables the analysis and release of information in seconds. Given these technologies, the IoT has a tremendous impact on offshore operations, enabling the integration of communication capabilities to sensors and managers [3,4]. The use of multiple hardware devices is unavoidable in this field. Their facilities for communicating and transmitting information related to the environment of the submarine cable and the submarine cable itself, such as disturbance (collision events), strain, and temperature level (thermal breakdown), are very important. While information about the sensing devices is sent to a local server, the rest of the information is stored in each module’s servo database for later analysis. As soon as the analysis is complete, IoT allows managers to receive commands and take timely intervention actions. Usually, to obtain low-latency responses, some data processing is executed in compute nodes close to sensing devices following the paradigms of edge computing [5]; communication is performed via wireless channels using open standards.

Integrated online monitoring systems for photoelectric composite submarine cables are mainly used in engineering application projects, such as offshore oil rigs and wind farms. These systems mainly include submarine cable monitoring in terms of temperature/strain, intrusion/collision, automatic identification system (AIS), and current-carrying ampacity analysis. Acquisition and remote data release are particularly important components of these systems.

With the improvement of industrial standards and specifications, the actual needs in different situations continue to pose challenging problems for the development and upgrading of system software, especially in harsh environments like offshore. Indeed, there is still a lack of in-depth discussions about dynamic system configuration and mass data transmission technology in the system software design process. In addition, for a single type of data, such as temperature, strain, and disturbance, the use of the application protocol data unit (APDU) defined in the IEC104 [6] for data transmission is very effective; however, it has certain limitations for transmitting a large amount of data in harsh environments.

The data applied to the submarine cable online monitoring system can be roughly divided according to their type and quantity, which can be further divided and transmitted based on their size. Among them, a single type and a small number of data can be transmitted through the type identification TypeID equal to 9 (normalized telemetry value), 11 (scaled telemetry value), 13 (short floating point telemetry value), and other APDU data packets in IEC104. Data types such as alarms and AIS are complex and include integers, characters, floating-points, and other data types. For this type of complex data information, this paper considers combining different types of data information into a string and then transferring it with a file according to the configured information object address.

### 1.2. Study Objectives

In practical applications, the command and control stations may need to be expanded and modified to suit different situations. Research on the system DC can effectively address this issue and enhance system compatibility and openness. In addition, the use of file transfer can solve the problem of releasing massive amounts of data in most telematics systems and adapt to complex data on various occasions. To overcome the challenges mentioned above, this paper provides the following contributions.

- The novel IoT architecture was designed with three layers: a data media layer, a control layer, and a servo application layer. The functions of these layers are to acquire, process, analyze, and release data in real-time. This IoT architecture makes it possible to evaluate data in order to improve its execution and release.
- The data types and files to be transferred are dynamically configured using IEC104 protocols to ensure an efficient and fast data release.
- The proposed IoT architecture was deployed in a real scenario in order to execute the data release of the photoelectric composite submarine cable and its environment. The experimental results obtained from the testing datasets during this deployment are presented using the proposed approach.

This paper is organized as follows: Related work on the fundamentals of IoT architectures and IEC104 protocols is highlighted in Section 2; Section 3 presents and describes the IoT architecture. In Section 4, the dynamic configuration for data release is described (key technologies and solutions). The engineering applications and analyses are presented in Section 5. Section 6, Section 7 and Section 8 present the validation of the proposed approach, discussion, and conclusion, respectively.

## 2. Related Work

This section covers the literature on IoT architectures in smart fields and marine environments and the application of DC techniques for data release.

### 2.1. IoT Architectures for Smart Fields

IoT architectures integrate security mechanisms, different communication protocols, and smart devices with limited resources [7]; in general, these types of IoT architectures are sustained by fog [8]. Recently, the use of IoT in smart factories has emerged as a means of propelling the digital revolution of production and empowering businesses to run more successfully, economically, and environmentally friendly [9]. Smart factories are gaining popularity, and the IoT plays a major role in enabling these factories [10]. The application of IoT architecture in advanced manufacturing has been proposed to increase the part production efficiency [11]. The Internet of Things for manufacturing is being researched to improve the efficiency of smart factory technologies in the production process for sophisticated parts [12]. A new secure cache decision system was introduced that follows the paradigm of fog computing and runs in a wireless network concentrated on smart buildings [13]. This system provides an efficient and secure environment for Internet browsing and data management. In addition, a variant of the optimistic concurrency control protocol in cloud-fog environments has been proposed and presented to improve the performance of these IoT architectures [14]. This mechanism not only significantly reduces communication time but also enables low-latency fog computing services for IoT applications. A new approach has been proposed to detect and share watermark image information in smart cities [15]. This generation method, which combines discrete cosine transformation with a convolutional neural network, has been successfully developed for security purposes.

### 2.2. Common IoT Architecture for Marine Environment

IoT-based offshore environment monitoring applications include several areas, such as ocean sensing and monitoring, ship monitoring, and submarine cable monitoring. These applications make use of various IoT system architectures, sensing, and communication technologies.

The IoT is capable of perceiving, thinking, and controlling environments by collecting, processing, and analyzing data from those environments. It is capable of making wise decisions that affect the external environment. IoT researchers in the academic literature have proposed various IoT system architectures. Five layers of IoT system architecture, including the perception and execution, transmission, data preprocessing, application, and business layers, have been proposed for monitoring and protecting the marine environment [16]. This IoT-based marine environment monitoring not only provides a good system for circulation and protection information but also reinforces services and preserves users’ privacy. The design and development of an IoT system must take these factors (physical topology and density) into account, as well as the deployment environment [17,18]. Multiple projects using IoT systems have been conducted to monitor the marine environment. An advanced, low-cost buoy system, tested in real-world conditions, has been designed for applications in the fields of ocean detection and monitoring [19]. Temperature, motion, vibration, and sound were used as the detection parameters. A VHF-based marine data acquisition and mapping system has been designed to detect vessels in secure areas at sea [20]. The detection parameters include temperature, depth, wind speed, and direction. For application in the field of water quality monitoring, real-time heterogeneous water quality monitoring was applied using inshore sensor buoys. It was deployed and tested at five sites on the River Lee, Ireland [21]. For application in the field of coral reef monitoring, remotely operated vehicles with water quality sensors were adopted using big data analysis [22]. ORP, pH, electrical conductivity, and audio/video were used as sensing parameters. For use in monitoring fish farms, researchers have developed a model based on temporal correlation that illustrates how ocean current velocity changes over time [23].

In the aforementioned literatures, IoT architecture is proven to be operational and reliable in different fields; however, these systems have faced some challenges due to the Internet going down, sometimes at crucial moments. This is considered a limitation in engineering applications; therefore, the device management unit in the proposed IoT architecture is developed using a suitable IoT agent.

### 2.3. IEC60870-5-104 Protocol to Evaluate Data Release

The IEC104 protocol is based on the development of models capable of acquiring information from input data and continuously releasing the final processed data. It is a set of general data transmission rules formulated by the IEC (International Electrotechnical Commission). Document [24] defined the data format and structure model of this protocol, and document [25] introduced the basic application functions and communication processes based on this protocol. Documents [26,27] introduce the definition and structure of the application service data unit (ASDU) for file transmissions. Researchers analyzed the IEC104 implementation method and successfully used it in a power system; the system worked well and had low latency [28]. Some researchers have introduced the application of the protocol in the SCADA (data acquisition, monitoring, and control) system; they provided design ideas for releasing automation software and made corresponding explanations for the transmission control protocol/internet protocol (TCP/IP)-based network programming [29]. During the experimental phases, a real positivity rate of 88% was achieved. A hardware communication module platform based on IEC104 was proposed and designed in the field of industrial communication [30]. The communication protocol of the system is well secured, and information is released in a timely manner. The precision rate of the release data is 98.6%; it is the one that has obtained the highest performance result. The authors of literature [31,32,33] introduced the application of IEC104 in photovoltaic power stations, substation auxiliary systems, and hydropower plants, respectively. The performance rates of these systems were 91.4%, 96.2%, and 93.2%, respectively. The remote control of these systems makes it possible to control separate locations over long distances from the centralized control room, which optimizes resource utilization for this task.

The application layer of IEC104 is combined with the transmission function provided by TCP/IP, and the communication is balanced; that is, both parties in the communication can initiate information transmission. Once the link is successfully established, the change information can be actively sent in addition to responding to the call response without waiting for a query. At the same time, the protocol defines the application protocol control information (APCI) in detail, as well as the purpose and usage of the three format messages (I, S, and U), and specifies important parameters such as the port number of the TCP connection.

**Figure 1:**
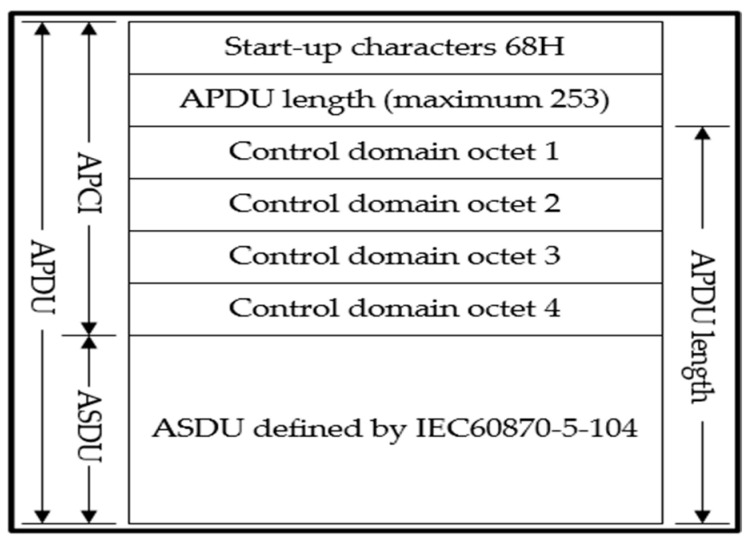
APDU definition of the tele-control supporting standard [6].

Figure 1 shows the definition of APDU in IEC104, which includes two parts: APCI and ASDU. APCI defines the starting point of the data stream, the length of the APDU, and the control domain. The control domain defines the control information that protects the message from being lost and repeated transmission, the start/stop of message transmission, the monitoring of the transmission connection, and other information. IEC104 extends the content of the ASDU in IEC101 and extends the corresponding part to define the type of information in the data transmission process, the reason for transmission, the public address, and information such as the address of the information object.

The IEC104 defines the information type identification used to transmit different messages in two directions: the process and system information of the monitoring direction (controlled station to the command station) and the process and system information of the control direction (command station to the controlled station). The main communication processes based on IEC104 are TCP connection establishment and station initialization, connection initiation, total call, time synchronization, total call of accumulated quantity (electricity, disturbance, etc.), remote control, and remote adjustment processes.

## 3. IoT Architecture for Data Release

**Figure 2:**
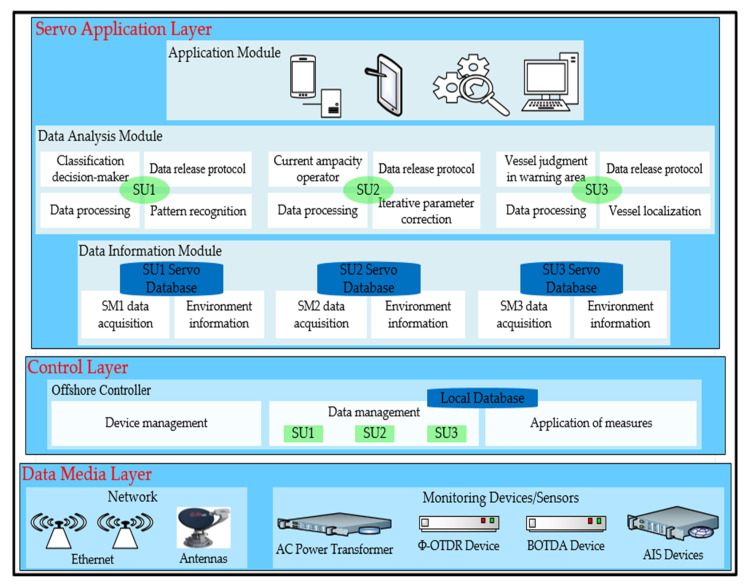
Scheme of the proposed IoT architecture for data release.

The IoT architecture for data release is designed to be scalable and flexible. It is mainly divided into three layers: the servo application, control, and data media. The data media layer enables data acquisition and transmission to the offshore controller (OC) in the control layer. The control layer receives the data and passes it to the servo application layer. The IoT architecture of the data release operates in two different phases: handling and transmission. During the handling phase, the data release architecture uses all the data collected from the monitoring devices/sensors to process them in an analysis module. During the transmission phase, the data release is ready to evaluate the recently handled data, analyze them, and execute the appropriate protocols to release them. With the exception of the data scanning unit and the unit of application of measures, all units operate equally in both phases. In particular, these two units do not occur during the handling phase. An architectural overview is shown in Figure 2.

### 3.1. Data Media Layer

The Data media layer is responsible for managing the devices/sensors used to collect and store data; it is close to the environment in which the data are collected on the offshore platform. They are mainly composed of devices that are responsible for collecting data from different sources and performing specific actions using actuators, which are used in conjunction with hardware through different protocols, such as the SPI and I2C protocols [34].

Three sensors, including Φ-OTDR, BOTDA, and AIS, are used in this IoT architecture. Phase-sensitive optical time-domain reflectometry (Φ-OTDR) is used for the disturbance, i.e., to detect and locate vibration signals from the optical fiber of the submarine cable, with a detection range of 20 km [35]. Brillouin Optical Time-Domain Analysis (BOTDA) is used to determine the temperature, strain, and current-carrying ampacity of submarine cables [36,37]. Its monitoring distance, positioning accuracy, and temperature measurement accuracy are about 15 km, 0.5 m, and 1 °C, respectively. The automatic identification system (AIS) is used to locate and determine the position of vessels [38,39,40]; its equipment is capable of monitoring vessels within a radius of approximately 40 km. Vessels arriving in the warning area can be effectively located using certain types of sensors or actuators, and a wide range of devices can be integrated. In addition, devices deployed in this layer typically have different power consumption. Therefore, it is necessary to integrate an AC power transformer to manage the power of each device because they work for different purposes in the same environment. Since the IoT architecture is an online monitoring system, an Ethernet switch is connected to the sensors and server to access the Internet. With the exception of the antennas (AIS and GPS antennas), which are installed on the roof of the oil rig, all other monitoring devices/sensors are in the control room. Another responsibility of this layer is to enable communication between the sensors/actuators and the control layer units.

### 3.2. Control Layer

The control layer is in charge of managing the tasks of the collected data. The OC is located in this layer and acts as an intermediary between the data media and the servo application layers. The aim is not only to achieve low latency in the communication between this layer and the data media layer, but also to perform control and management tasks on the devices. This layer contains three units: device management, data management, and control management (application of measures).

#### 3.2.1. Device Management Unit

This unit is developed using a suitable IoT agent, which is a generic enabler for managing backend devices. It is responsible for managing devices in the data media layer; it manages the connectivity of all devices in the architecture and ensures their proper functioning. If new devices are connected to the IoT architecture, they must communicate with the device management unit to obtain their configurations.

When the devices are set up, the device management unit provides each device with a unique identifier and records its activities in the local database. In addition, there is information about the location of the device and its network configuration. This information allows the device management unit to periodically check and verify that communication with the device is configured correctly and that information is sent accordingly.

#### 3.2.2. Data Management Unit

It is responsible for managing and storing data from monitoring devices and sensors. This unit has three subunits, including Subunit 1 (SU1), Subunit 2 (SU2), and Subunit 3 (SU3). SU1, SU2, and SU3 are dedicated to the data collected from the Φ-OTDR, BOTDA, and AIS devices, respectively. When each of these devices collects data, the data are sent to the OC, where they are received by the data management unit. This has two main functions: (1) It filters and stores data in the local database. (2) It synchronizes the local database, which is in the control layer, with the servo database in the servo application layer. SU1, SU2 and SU3 each have their own database in the local database.

Usually, data collected by sensors includes false and nuisance alarms that must be eliminated; hence, the need to filter data. After filtering, this unit selects the data that will be finally transferred to the servo and ensures that they are of good quality. Regarding data synchronization, data from the OC is always sent to the servo application layer. If there is a loss of information in the OC, the data management unit can retrieve that information from the servo database and store it in the local database.

The device management unit is implemented using an appropriate IoT agent, which is a generic enabler for primary device management. This component acts as a gateway to route information to the servo application layer using specific topics defined in the message queue telemetry transport server, which is the central hub for the publish/subscribe model.

#### 3.2.3. Application of Measures Unit

It is responsible for controlling and monitoring the execution of the commands in the actuator. These commands produce effects that eventually correct the abnormalities measured by the sensors. The OC monitors the sensor data and can autonomously implement corrective measures based on the decisions provided by the servo application layer.

Operators registered in the application module apply the corrective measures; they are spread all over the system until they reach the OC, where they are transmitted to the actuators. The local database stores the values and defines their range, which is where the measurements made by the monitoring devices/sensors should be included. For each sensor, the measurements should be within the normal measurement range; otherwise, the OC can take measures independently to correct this case. In addition, corrective measures are based on the results declared by the analysis module.

Policies are defined by operators to enable corrective measures. These policies are stored in the control layer (local database) and the servo application layer (servo database).

### 3.3. Servo Application Layer

The servo application layer is responsible for processing, displaying, and transmitting data to the command and control station. As the highest layer of the architecture, the servo application layer is always waiting to monitor the data after a connection is established. It consists of three modules: data information, data analysis, and application.

#### 3.3.1. Data Information Module

This module receives data from installations deployed on offshore platforms. Not only does it manage and aggregate this data, but it also manages information about the submarine cable environment. This module consists of two units: data acquisition and environment information.

- Data acquisition Unit: It receives information from the OC deployed in the facility and is responsible for storing it in the corresponding servo database. This information includes data from the sensor and OC itself. In addition, each OC is identified by a unique identifier allocated to it during installation, which links the data stored in the database.
- Environment information unit: The purpose of this unit is to coordinate and aggregate the data collected by each sensor located in the data acquisition unit and store it in its corresponding servo database. It is responsible for extracting environmental information from all these data and making it available for subsequent analysis in the data analysis module. Indeed, the data received from the sensors and provided by the data acquisition unit are available to the data analysis module via the servo database. Since each sensor has its own corresponding servo database, the system continuously updates the data acquired in each servo database.

#### 3.3.2. Data Analysis Module

This module contains procedures for extracting knowledge from the data information module. It can be seen that this module has three departments, like the lower module, and each department is in charge of the data from its sensors. The purpose of this module is to apply appropriate models and protocols to serve on-demand results to the application module. The units of each department are oriented to organize the data to be used with the designed algorithms and protocols.

With the exception of the data processing and data release protocol units, the units in each department are different since their sensors have different functions. The work process in this module is as follows: The data are extracted from the servo database, which contains all the necessary information; these data pass through the data processing unit to be prepared for use in subsequent units. The next two units implement the models used in this department to obtain the final treated data that will be released in the final unit of the department. The final unit contains the design scheme for the remote data release function. The data are released with the corrective measures defined in the servo database.

- Data processing unit: This unit processes all the data stored in the servo database, scales the continuous characteristics present in the datasets, and encodes the class variables following a model appropriate for the problem under study. In addition, it not only provides the possibility to choose these datasets using an approach, but also allows the datasets to be divided while preserving the time consistency of the data. This unit has the same function in each of the three departments; the first, second, and third departments contain SU1, SU2, and SU3, respectively.

**Figure A1:**
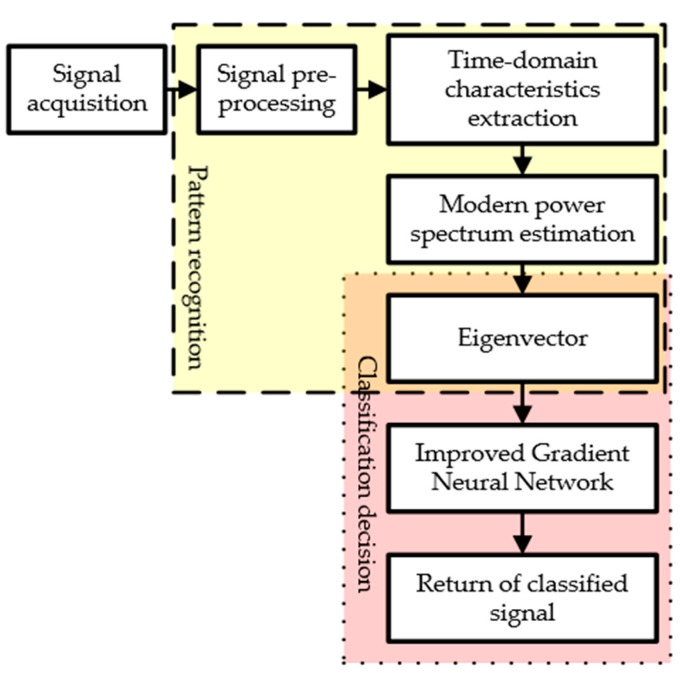
Architecture of the hybrid model for optical fiber vibration signal processing.

**Figure A2:**
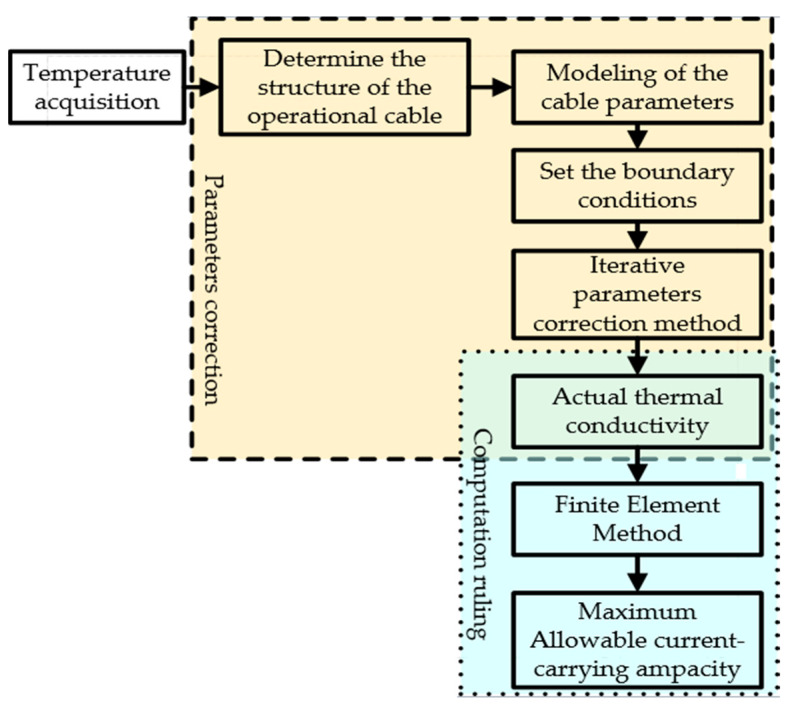
Structure for calculating the current-carrying ampacity.

Once the data are prepared in the data processing unit, they pass through the next two units to be processed according to the algorithm designed and implemented in these units. In the three departments, these two units succeeding the data processing unit are totally different since each department deals with different problems. For example, (1) the first department deals with disturbances (collision events/malicious attacks). The second unit in this department is the pattern recognition algorithm, which is composed of time-domain characteristics extraction and modern power spectrum estimation (MPSE) [41]. This enables the detection and recognition of disturbance events along the submarine cable. The third unit is the classification decision-maker, which is designed by the improved gradient neural network (IGNN) and used to classify the type of disturbance. A flowchart of the MPSE-IGNN is presented in Figure A1 (see Appendix A). (2) The second department deals with temperature, strain, and current ampacity. The second unit in this department is the iterative parameter correction based on the principle of the chord method (IPCM) [42]. This method enables the actual temperature to be within the allowable error range, thus making it possible to determine the correct current ampacity using the finite element modeling (FEM) method in the third unit. A flowchart of the IPCM-FEM is presented in Figure A2 (Appendix A). (3) The third department deals with the position of vessels in the warning area. In the second unit of this department, vessel localization using the interactive multiple model (IMM) algorithm with the time difference of arrival (TDOA) and frequency difference of arrival (FDOA) of vessels is implemented [43]. The third unit of this department contains the method of vessel judgment in the warning area; a short message stating that “You are entering the precautionary area of the submarine cable, an anchorage cannot be allowed in this zone” is automatically sent to vessels that are about to enter the warning area. It is worth mentioning that in the third unit of each department, the alarm function will always trigger if an anomaly is detected. For example, if there is an aggressive attack on the cable, if there is a rise in the cable temperature, which affects its current ampacity, or if a vessel is entering the warning area for anchorage.

**Figure 3:**
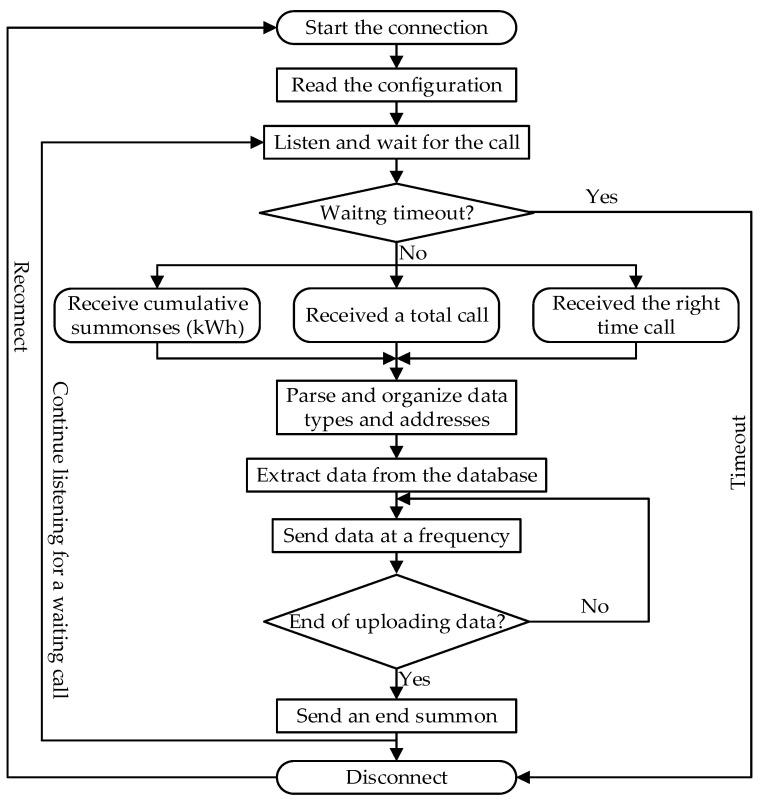
System operation process.

**Figure A3:**
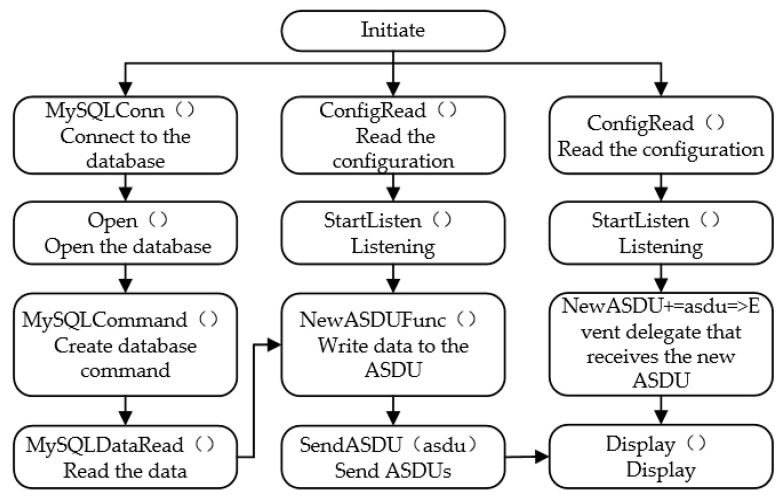
Design of the main thread in the system.

- Data release protocol unit: This unit operates in the same way in each of the three departments. It completes the underlying DC and remote data release functions based on IEC104. It parses the data received by the previous unit and simultaneously performs the associated database operations.

1. Operation process: After starting the connection, the system starts to read the configuration; at this time, the controlled station is monitored and waits for the call. If the call message command from the command station is not received within the specified time range, it will be judged as “waiting timeout”, and the connection must be reconnected. When the controlled station receives the call message command sent by the command station, it begins to analyze it and organize the data type and address to extract the corresponding data from the database and send it to the command station at a certain frequency. After all the data are sent, the controlled station sends a message command to end the call and terminate the data transmission. This process is displayed in Figure 3.

2. Main thread design: The flowchart is shown in Figure A3 (Appendix A). The database connection thread mainly realizes the database connection and related operations, and the data sending and receiving display thread mainly realizes the data interaction between the command and controlled stations. The communication parties start to read the configuration after starting the connection, and then enter the waiting state for monitoring. When the communication party receives the data read from the database, it starts to pack and organize them before finally sending them to the other party for analysis and display.

In the data release protocol unit, there is a mapping relationship between the information object, address, and storage location in the servo database. When receiving a call to start transmitting data, the controlled station extracts the ready-to-send data, fills it into the APDU, and then gives it the corresponding address for transmission. Taking into account storage capacity and real-time issues, each information in the database will also be periodically refreshed and deleted.

#### 3.3.3. Application Module

This module is responsible for providing operators with different operational and service applications. The applications that can be found in this module can be on desktops, laptops, and mobile phones; web applications can also be added. Upon a specific request, the application module communicates with the data release protocol unit to obtain and display the resulting data.

## 4. Dynamic Configurations for Data Release

### 4.1. Monitoring Software System

Indeed, project files and workspaces are “ini” files that contain LabWindows/CVI configuration information for the program. The application uses the configuration information for the mid-density segment of parameter.ini. This software boasts a robust library of functions, many of which are effectively wrapped, eliminating the need to write them from scratch. Table 1 shows the indicators of the application environment.

#### 4.1.1. Implementation of the System Protocol Layer

In the implementation of the system protocol layer, the following data structures, that is, APCIParameters, ApplicationLayerParameters, CauseOfTransmission, ASDU, Type ID, and the information body elements, are defined based on the EIC104.

#### 4.1.2. Server-Side Parameter Configuration

This configuration was applied to the relevant parameter settings. It secures the transmission control mechanism, sets the byte length of each component and the maximum length of the ASDU, sets various states of client connections, sets different modes of the client connection server, and sets the host name, port number, maximum length of the ASDU queue, and maximum number of connections. The specific configuration is as follows:

- APCIParameters configuration: The maximum number of APDUs in “I-format” that are not acknowledged by the sender (k = 12); the receiver receives up to the number of APDUs in unacknowledged “I-format” (w = 8); the network timeout period for establishing a connection (t0 = 10); the timeout period for sending or testing the APDU (t1 = 15); the timeout period acknowledged when the receiver has no datagram (t2 = 10), with t2 < t1; and the timeout for sending acknowledgments when the channel is idle for a long time (t3 = 20).
- ApplicationLayerParameters configuration: The number of bytes identified by the type is 1 (sizeOfTypeId = 1); the number of bytes of a variable structure qualifier is 1 (sizeOfVSQ = 1); the number of bytes for the reason for transmission is 2 (sizeOfCOT = 2); the number of bytes of the public address is 2 (sizeOfCA = 2); the number of bytes of the address of the information object is 3 (sizeOfIOA = 3); and the maximum length of ASDU is 249.
- ClientConnectionEvent configuration: The connection is open (OPENED); the connection enters the active state, in which the two communicating parties can exchange data (ACTIVE); the connection enters an inactive state, in which the communicating parties cannot interact with each other (INACTIVE); and the connection is closed (CLOSED).
- ServerMode configuration: Single-connection activation mode (SINGLE_REDUNDANCY_GROUP) and multi-connection activation mode (CONNECTION_IS_REDUNDANCY_GROUP).
- Connection control parameter configuration: The local hostname is “0.0.0.0” (localHostname = “0.0.0.0”); the port number is 2404 (localPort = 2404); the maximum length of the ASDU queue is 1000 (maxQueueSize = 1000); and the maximum number of connections is 10 (maxOpenConnections = 10).

Two different working modes, including single-connection activation mode and multi-connection activation mode, are designed to facilitate the control of the data communication connection and effectively improve the efficiency of data transmission. In the multi-connection activation mode, multiple connections can be active at the same time, and all active connections can be used for data transfer. In the single-connection activation mode, only one connection can be active, and the others are in the pending state.

### 4.2. Dynamic Configuration of Command and Controlled Stations

DC is applied based on the type and amount of data. Table 2 shows the types and amounts of data that can be found in oilrig offshore systems. The DC of the command and controlled stations is a very important link in the remote release system, which solves the constraint problem of these stations for each physical amount of data, data type, and address allocation; thus, the data transmission of the two communicating parties has rules to follow.

#### 4.2.1. Dynamic Configuration of a Single Type of Data

A single type and a relatively small amount of data information objects can be transmitted through the APDU defined by the IEC104 specification, for which it is necessary to design a mapping relationship between the information object, address, and storage location in the servo database. The designed mapping scheme is shown in Table 3; the amount of information depends on the size.

In the servo database SeaCableMonitor (storage location: D:MySQLSeaCableMonitor), there are three tables: Current, ProtectiveLayer, and State. These tables are used to store three types of data information, namely electric energy, cladding monitoring, and equipment status; each table contains several records (rows) and fields (columns). The record displays the cable data information of each channel; the field stores the cable number, location, time, and amount of information at a certain period of time; and the monitoring equipment stores the data collected at a certain period of time under the corresponding field of each table. When the handler receives a data request, it removes the fields from the corresponding table and assigns the address of the information object, which is packaged and sent to the command station.

#### 4.2.2. Dynamic Configuration of File Transfer

The problem that the DC of file transfer needs to solve is the storage location of the generated data file and the establishment of the mapping relationship between the section name of the file and the information object. The files are stored in the D:\SeaCableMonitor\Files\directory.

Table 4 shows the mapping relationship between the file section name and information objects. Considering multiple features, such as scalability, interoperability, and visualization, this paper selects a simple data storage language, XML (Extensible Markup Language), to implement the above dynamic configuration. When the command station or the controlled station needs to be extended or changed, only the configuration file needs to be modified without dealing with the complicated source code. The code below (Algorithm 1) shows the configuration process of an information object and its address.
**Algorithm 1.** Configuration algorithm<MsgConfig>        <MsgType> Information type         < TypeId Code=’9’> Normalized telemetry </Code>         ……         </MsgType>         <MsgAddr> Information object address         <Current_Begin addr=’0x1’> Power information start address </Current_Begin>         <Current_End addr=’0x5000’> Energy information end address </Current_End>         ……         </MsgAddr> </MsgConfig>

### 4.3. File Transfer Application

The range of information body addresses defined in IEC104 is 1~65,534, that is, the system can hold a maximum of 65,534 data, and the maximum length of an ASDU is 249 (APDU maximum = 255 minus startup and length octets). Thus, the number of bytes of the information body is likely to exceed the maximum length of the ASDU, and it is impossible to carry out massive data transmission. In addition, data information such as alarms and AIS contains various data types such as integers, characters, and floats; therefore, the number of bytes of the information body in the data transmission process is not fixed, and address allocation cannot be performed. Therefore, this paper considers the use of file transfer schemes to overcome this problem.

File transfer is used when the number of bytes of the information body in the tele-motion system exceeds the maximum length specified by the ASDU, and the information is transmitted to the destination in a segmented form. The file structure is the same in both directions (command to controlled stations and vice versa) and is transferred in both directions in the order of sections. Usually, a file contains several sections, and one section contains many other sections. In a telematics system, files can be transferred in both directions. Table 5 shows the type identification for file transfer defined by IEC104.

File transfer in the monitoring direction (from the controlled station to the command station) is mainly used to notify the command station that an event has occurred and a large amount of data has been logged. Before starting the file transfer, the controlled station must send a file directory “DIRECTORY PDU F_DR” to the command station and inform the command station in the form of PDUs of the number of files, the login time, and the event type. After receiving the directory PDU, the command station sends a SELECT_FILE PDU F_SC to notify the controlled station to start selecting files. At this time, the controlled station returns the FILE_READY PDU F_FR to indicate that the file is ready and can be called.

Next, the controlled station uploads the file section by section to the command station according to the file structure. After all file transfers are completed, the controlled station deletes the data records of the files that have been successfully transferred to create space for new files. File transfer in the control direction (command station to controlled station) is mainly used to download parameter tables or programs. The command station arranges the type, quantity, and scale of the data file to be transmitted without the need to transfer the directory. Table 6 and Table 7 show the definitions of the file transfer directory ASDU (TypeID = F_DR_TA_1) and segment ASDU (TypeID = F_SG_NA_1) defined in IEC104, respectively.

It must be mentioned that: (1) The information object address during the file transfer does not represent any information and is uniformly set to 000000. (2) The maximum length of the segment is between 234 (the maximum length when the link field and data unit identifier match the information object address) and 240 (the minimum length when the link field and data unit identifier match the information object address).

#### 4.3.1. Transfer of Massive Amounts of Data

For example, the system server needs to release one million temperature data points at a time because it is floating-point data, the total length is 4 million bytes, and the length of the definition section is 104 B. The number of sections contained in the file is

(1) $$\frac{4000000}{10000}=400$$

If the length of the defined segment is 100 B, the number of segments contained in each section is

(2) $$\frac{10000}{100}=100$$

Therefore, the file transferred by the system contains 400 sections, each with a length of 10 KB, and each section contains 100 segments, each with a length of 100 B. The system server transfers the file data to the client in the order of the segments.

#### 4.3.2. Transfer of Complex Types of Data

The complex types of data in our integrated online monitoring system include AIS data and alarm data. For this type of data, this paper considers combining different types of data information into a string and then transferring it with a file according to the configured information object address shown in Table 3.

File transfer of AIS data: AIS data includes the MMSI identification number, device type, latitude and longitude, speed, heading, and other data. Therefore, in this paper, the structure is first used to encapsulate these data into a memory block, corresponding to a section in the file; such a section represents the AIS data information. The data are then sent out in segments. The AIS information is of each data type, and the number of bytes it allocates is shown in Table 8. AIS data information accounts for 50 bytes, that is, the length of a section is 50 bytes. According to the structural definition of file transfer, the AIS data information is divided into 10 segments, and each segment accounts for 5 bytes; that is, the length of a segment is 5 bytes.

- File transfer of alarm data: Table 9 shows the configuration of the alarm data type and the number of bytes allocated. It can be seen from the table that the alarm data accounts for 80 bytes; these data are divided into 10 segments, and each segment accounts for 8 bytes.

## 5. Engineering Application Case

**Figure 4:**
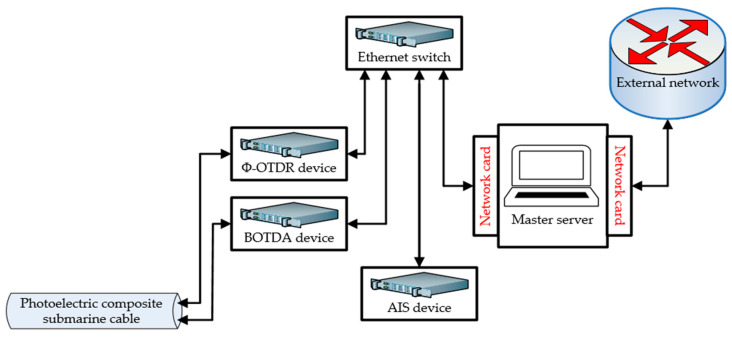
Photoelectric composite submarine cable online monitoring system.

The proposed approach was deployed in a real-world scenario for more than one year at the port of Zhangjiagang, which is part of the East China Sea offshore wind energy project. The monitoring devices/sensors and photoelectric composite submarine cables used in this application are all functional. The objective of this application is to evaluate and release data based on the information collected by the sensors deployed on the offshore platform. Figure 4 shows the general composition of the integrated online monitoring system, which is mainly composed of a submarine cable, data acquisition equipment, and other auxiliary equipment, including an Ethernet switch and server.

This section focuses on the operations executed in the units of the data analysis module, as they are responsible for running the models to process and release the data.

### 5.1. Experimental Setup

**Figure 5:**
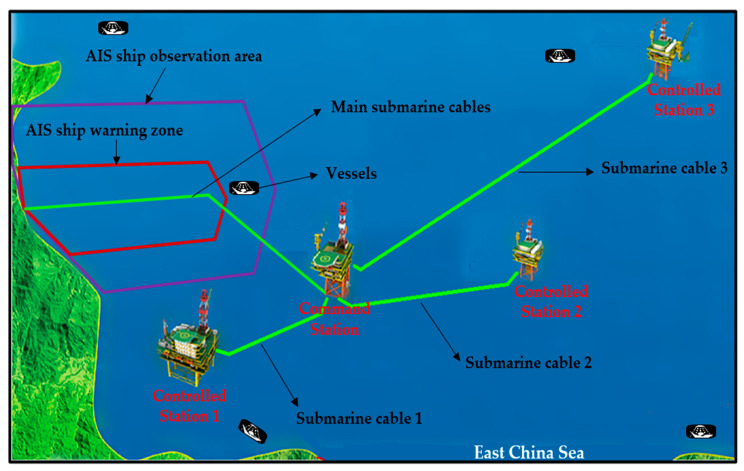
Block diagram of experimental system.

There are four oilrig stations, including one command station and three controlled stations. Each controlled station is connected to the command station by means of a submarine cable. Controlled stations 1, 2, and 3 are supplied with energy power by a 3 km submarine cable 1 (22 kV three-core XLPE cable), a 5 km submarine cable 2 (9 kV single-core XLPE cable), and an 11 km submarine cable 3 (35 kV three-core XLPE cable), respectively. The main submarine cable is a 220 kV three-core XLPE submarine cable that connects the command station to the offshore wind farm. The main submarine cable is a high-voltage cable, submarine cable 2 is a low-voltage cable, and submarine cables 1 and 3 are medium-voltage cables. The experimental configuration of the system is depicted in Figure 5.

In the four oilrig stations, different operational applications for human-machine interfaces are deployed. In addition, all the stations have the same monitoring devices/sensors deployed in their data media layer, and the Internet antennas are placed on the roof of the command and controlled stations. The system collects 250 sets of data samples in real-time, including 80 frames from BOTDA devices, 120 frames from Φ-OTDR devices, and 50 frames from AIS devices; the length of each frame is 0.28 s. Over the course of more than a full year, which consists of four seasons: summer, fall, winter, and spring, data were collected, processed, and released on an ongoing basis.

**Figure 6:**
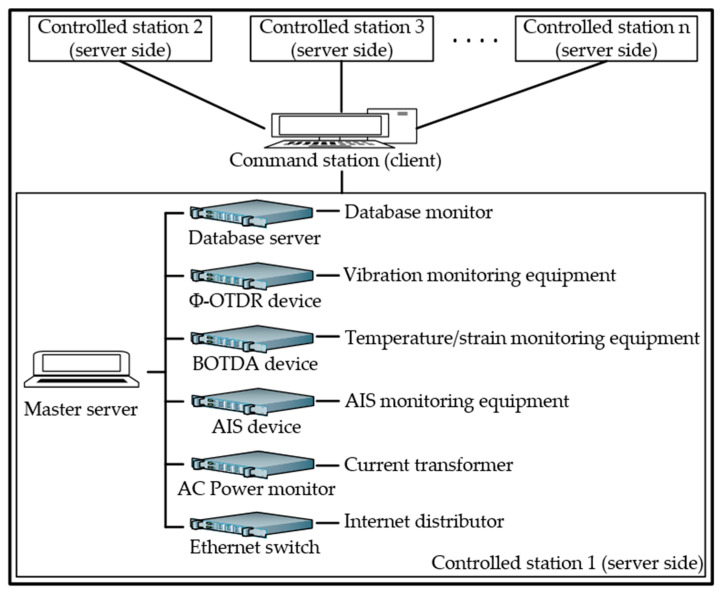
Overview of the integrated online monitoring system.

The general structure of the communication system is depicted in Figure 6. The monitoring equipment of the controlled stations (server side) stores the collected data in the local database and waits for a data call from the command station (client side). The monitoring devices and sensors are all registered in the offshore controller located in the control layer via the device management unit. Once these sensors are registered, they are ready-to-send data, which are managed by the data management unit. The data sent to the data management unit are filtered and stored in the local database. As we encounter some challenges because the Internet is unstable and sometimes cuts off, this communication is carried out using the IoT agent provided by the offshore project. This makes it possible to have the Internet at any time; as a result, data can be collected, processed, and released continuously.

### 5.2. Experimental Verification

These verifications consist of evaluating and releasing EED1 and DD3 to the command station at certain periods of time. EED1 is the electrical energy data of submarine cables 1, and DD3 is the disturbance data of submarine cables 3. In addition, the data of the vessel arriving at the warning zone are treated, and then the controlled warning message is released to this vessel from the command station. These verifications are more focused on the data analysis module. Thus, as detailed in Section 3, the corresponding device in the data media layer collects the data. After the rules are applied in the control layer, this data will be sent to the appropriate servo database in the servo application layer for treatment, analysis, and release.

#### 5.2.1. EED1

The process of treating and releasing these data is as follows:

- Data processing unit: Once the data sent by the OC are stored in the SU2 servo database, the next step is to adapt them for use in the designed model IPCM-FEM; this adjustment is performed in the data processing unit. Since the data are collected at different time intervals, preliminary data processing is required to prepare the data before moving on to the next unit. For example, the electrical energy of an XLPE submarine cable depends on its insulation temperature and the electrical resistance of its conductor [44]. The temperature of the underwater soil generally varies between 14 °C and 29 °C depending on the season, and this temperature is very important to know because it influences the calculations to determine the electrical energy. Thus, the insolation temperature of the cable is always collected by the BOTDA device, regardless of the season. However, at the time the experiments were carried out, the temperatures of the seawater and cable insulation were about 20 °C. The convective heat transfer coefficient between soil and seawater is 200 $\left(W/m^{2}.℃\right)$. All this information is stored in a database and is available at any time.

In addition, this unit created the time series to run the implemented model; this time series is generated taking into account the submarine cable 1 information from the moment the BOTDA device starts working. After all the data are collected, this unit generates the training and test datasets that are already available. Finally, the data processing unit scales the data for use in the IPMC model.

- Iterative parameter correction unit: The IPCM used to correct errors that may occur in the calculation of electrical energy is designed and implemented in this unit. In the temperature field modeling, physical performance parameters such as heat capacity and thermal conductivity K of the submarine cable are defined in this unit. For example, T and $T_{0}$ are respectively the final and actual temperatures of the cable insolation, collected in a specific time interval. T is a function of K (T=f(K)), and ε is the error limit. If ${T-T}_{0}\leq \varepsilon$, then K is the accurate value; if ${T-T}_{0}>\varepsilon$, then the value of K should be corrected. When ${T-T}_{0}$ is not within the error limit, the IPCM is applied using the chord method, which describes how to modify the parameters through the function values of each node obtained by modeling [45]. The formula applied to correct the parameters in this unit is as follows:

(3) $$K_{i+1}=K_{i}-\frac{f\left(K_{i}\right)-T_{0}}{f\left(K_{i}\right)-f\left(K_{i-1}\right)}\left(K_{i}-K_{i-1}\right)$$

where $K_{i+1}$ is the corrected parameter value, $f\left(K_{i}\right)$ is the modeling temperature value at $K_{i}$, $T_{0}$ is the actual temperature value. Since the parameters are within the allowable error range, it is only necessary to stop the iteration when ${f\left(K_{i}\right)-T}_{0}\leq \varepsilon$. Thus, the cable ampacity can be accurately calculated.

- Current ampacity operator unit: The FEM was implemented in this unit. This unit defines not only the geometric model of submarine cable 1 according to its structural parameters and laying environment, but also the physical performance parameters of its different materials. This allows its meshing in order to establish the finite element model. For example, the range of the ambient temperature is $\left[T_{1},T_{2}\right]$, the temperature interval is ∆T, and the range of convective heat transfer coefficient is $\left[h_{1},h_{2}\right]$ and the interval is ∆h. Set the initial ambient temperature T as $T_{1}$ and the initial convective heat transfer coefficient h as $h_{1}$. Set the initial value of the cable working current $I_{0}$ to I. Input the working current $I_{r}$ of the cable, the ambient temperature T, and the convective heat transfer coefficient h in ANSYS modeling software (Version 2022). Then, obtain the temperature field distribution as well as the cable insolation temperature t and conductor temperature $T_{c}$. Submarine cable 1 has a rated current $I_{r}$ of 704A, which is confirmed by the steady-state current calculation formulated by the IEC 60287 standard [46]. Based on the result obtained from the previous unit, the parameters go through multiple evaluations before the periodic load factor M is generated by the ANSYS modeling to determine the final electrical energy $I_{z}$. Its equation is:

(4) $$I_{z}=M\times I_{r}$$

When performing the calculations, the value of M was 1.24. After computation, the final electrical energy $I_{z}=872.96\;A$ is ready to be released.

- Data release protocol unit: Data release is executed in this unit following a specific logic. Knowing that electrical energy is a single type of data, the transmission of such data is as follows: Considering the transmission of a single-energy data for submarine cable 1, the configuration parameter information is set as shown in Table A1 (See Appendix A). After the system starts reading the configuration and waits for the data to be summoned, Wireshark software (Version 2023) is used to analyze the network capture of the communication process to obtain the packets received by the command station, as shown in Table A2 (See Appendix A). As shown in Table A2, the code of the transmitted normalized telemetry value is 10a1 (hexadecimal); to obtain the specific transmission value, further calculations are required using the given parameters. The transmission code is as follows:

(5) $$\frac{4257\left(10A1H\right)}{32768(80000H)}$$

#### 5.2.2. DD3

The process for handling and transmitting data in this department is as follows:

- Data processing unit: As mentioned earlier, when the data sent by the OC are stored in the servo database, they are adapted in the data processing unit for use in the designed MPSE-IGNN model. Since the data are collected under different conditions or at different time intervals, it is necessary to prepare them in this unit before they enter the next unit. For example, when the Φ-OTDR device detects the vibration of the optical fiber of the cable, it indicates that there is a collision event with the cable. The vibration data of the optical fiber of the cable are always collected for processing in order to determine the magnitude and position of the collision event. At the time of the experiment, submarine cable 3 was struck with a thrust force of about 45 Newton by means of a 100 kg iron hammer; this data corresponds to the second class, which can be verified from Table A3 (See Appendix A).

Once the data are collected, this unit generates the training and test datasets before scaling them for use in the MPSE model.

- Pattern recognition unit: The MPSE is used in this unit, which consists of the power distribution of the extracted signal features in the time and frequency domains. For example, the instantaneous energy (U) and instantaneous threshold rate (V) are used to extract signal features from the power distribution in the time-domain through the following equations:

(6) $$U=\sum_{i=1}^{N}X_{i}^{2}(i=1,2,\ldots ,N)$$

(7) $$V=\frac{1}{2}\sum_{i=2}^{N}\left\{\left|sgn\left(X_{i}-\delta \right)-sgn\left(X_{i-1}-\delta \right)\right|+\left|sgn\left(X_{i}+\delta \right)-sgn\left(X_{i-1}+\delta \right)\right|\right\}$$

where $X_{i}$ with i=1,2,…,N is the data sample, δ is the threshold, and $sgn(x)=\left\{\begin{array}{c} \;1,\;x\geq 0\\ -1,x<0 \end{array}\right.$.

The multipolar autoregressive model is used to extract signal features from the power distribution in the time-frequency domain via (6).

(8) $$P_{x}\left(k\right)=\sigma^{2}\times {\left|T(z)\right|}^{2}=\frac{\sigma^{2}}{{\left|1+\sum_{k=1}^{d}a_{k}z^{-k}\right|}^{2}}$$

where $a_{k}$, d, $\sigma^{2}$ and T(z) are the order coefficient, the order of the model, the variance and the transfer function, respectively. Together, both features (in the time and frequency domains) form an eigenvector, which is used for training the next unit.

- Classification decision-maker unit: The IGNN is implemented in this unit to determine the types of events corresponding to the eigenvectors formed in the previous unit. The eigenvector is normalized and input into the multiclass IGNN classifier to initiate the extent and position of the collision event in real-time. To evaluate the similarity between the predicted values and eigenvalues, the following equation is used:

(9) $$L\left(\hat{y},y^{*}\right)=-\sum_{i}^{Nclass}y_{i}^{*}ln\left({\hat{y}}_{i}\right)$$

where $\hat{y}$ is the predicted label vector and $y^{*}$ is the eigen-label vector. After evaluations, the classified signal $\hat{Y}$ is obtained and ready to be released. For example, during the experiment, the second class of collision events was emitted as a classified signal. The collision occurred 0.17 km away from the Φ-OTDR device connected to the optical fiber of the cable, and the probe response time was about 0.6 s.

- Data release protocol unit: The logic for releasing these data includes the transmission of a disturbance data file for submarine cable 3. Table A4 (See Appendix A) provides its parameter configuration information, from which we can see that the length of the file is 750 KB, the length of the section is 7680 B, and the length of the segment is 120 B. The number of sections and segments is determined as shown in (10) and (11), respectively.

(10) $$\frac{750\times 1024}{7680}=100$$

(11) $$\frac{7680}{120}=64$$

Similarly, using Wireshark software to perform network packet capture analysis on the disturbance data file transmission process, the message can be obtained (only the first paragraph of the first section of the transmission is displayed). Table A5 (See Appendix A) shows the segment packets received by the command station with type ID = 7d in file transmission. According to the configuration information, it can be parsed that the command station has received the file data information with file name 1, section name 1 (disturbance), and segment content 1.

#### 5.2.3. Vessel Arriving at the Warning Zone

The process for the handling and transmission of the data in this department is as follows:

- Data processing unit: When the data are stored in the servo database, they are accommodated in this unit for use in the designed model IMM-TDOA/FDOA. Once the data is inserted into this unit, it is scaled before being used in the IMM-TDOA/FDOA model.
- Vessel localization unit: The IMM-TDOA/FDOA model is implemented in this unit using the following equations:

(12) $$∆t_{i}={t_{i+1}+t_{i}=r}_{ti}\left(x,y,z\right)+{∆n}_{t}$$

(13) $$∆f_{ri}=r_{fi}\left(x,y,z\right)+{∆n}_{f}$$

where $∆t_{i}$, $∆f_{ri}$, ${∆n}_{t}$ and ${∆n}_{f}$ are the TDOA of the adjacent signals received by the vessel, the FDOA of the adjacent AIS signals, the difference of noise between the two time measurements and the difference of noise between the two frequency measurements, respectively. $t_{i}$ is the time spent by the ith AIS signal transmitted from a satellite to vessels. Since the vessel’s position using the IMM algorithm is $\hat{L}={\left[\hat{x},\hat{y},\hat{z}\right]}^{T}$, the localization model IMM-TDOA/FDOA is:

(14) $$\left[\begin{array}{c} ∆T\\ ∆F\\ \hat{L} \end{array}\right]=\left[\begin{array}{c} r_{t}\left(x,y,z\right)\\ r_{f}\left(x,y,z\right)\\ {\left[\hat{x},\hat{y},\hat{z}\right]}^{T} \end{array}\right]+n_{c}$$

where ∆T and ∆F are, respectively, the TDOA and FDOA measurement vectors obtained by synchronization technique; $n_{c}$ is the measurement noise matrix having the signal propagation velocity. x,y and z are the geodetic coordinates of the vessel with $x=R_{N}cos\lambda cos\varphi$, $y=R_{N}sin\lambda cos\varphi$ and $Z=R_{N}\left(1-e^{2}\right)sin\varphi$ where $R_{N}=\alpha /\sqrt{1-e^{2}{sin}^{2}\varphi }$ is radius of curvature in prime vertical (α = 6378.137 km represents the earth’s equatorial radius determined by WGS-84, and $e^{2}$= 0.00669437999013 represents the square of the first eccentricity). After the vessel is localized, the data can move to the next unit.

- Vessel judgment in the warning area unit: The vessel position judging model is implemented in this unit to determine whether a short message should be sent to them. For example, if $P\left(X_{i},Y_{i}\right)$ is the point where the vessel is located, the following equation is applied:

(15) $$Z\%2=\left\{\begin{array}{c} 0\;\;\left(X_{i},Y_{i}\right)Not\;entering\;the\;warning\;area\\ 1\;\;\left(X_{i},Y_{i}\right)\;\;\;\;Entering\;the\;warning\;area \end{array}\right.$$

Z%2 is the number of intersections Z to 2, which takes the rest, and then uses the parity of that rest to judge whether the vessel enters or not in the warning area. Since the vessel is considered to be entering the warning area, the system should be ready to send a short message to the targeted vessel.

- Data release protocol unit: AIS contains several types of data; therefore, the number of bytes in the data transmission process is not fixed, and address allocation cannot be performed. Therefore, only the file transfer scheme is implemented in this unit. As the AIS contains complex data, its data release follows the following logic. The system defines two AIS devices and automatically allocates the serial port data processing threads. Each serial port data processing thread allocates two thread security queues for transmitting and receiving the data. The INI configuration file is presented in Table A6 (See Appendix A).

AIS decoding is performed in strict accordance with the provisions of the International Telecommunication Union ITU-1371 [47,48]. Secondary development of AIS equipment is carried out so that the system can accept short messages and send instructions from the server to the targeted vessel. The short message transmission format is as follows: -$XNMSG, message ID, nine-digit code, message type, statement content * check code. Message ID: It is a “three digits” that represents the ID of the message being sent. The types of short messages are shown in Table A7 (See Appendix A). Check code: It is a “two-digit hexadecimal number”, that is, the string XOR check between $ and *.

### 5.3. Data Display Software Overview

**Figure A4:**
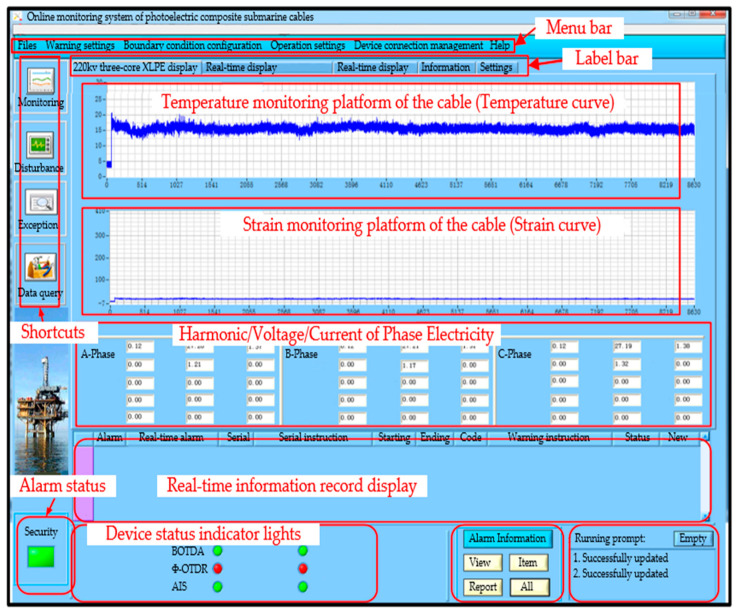
Block diagram of monitoring software system.

The user must enter a password in order to access the settings page before using the system. Figure A4 (See Appendix A) shows the main interface of the integrated online monitoring data release system at the command station. It includes the menu bar, label bar, shortcut mode, alarm processing bar, alarm status indicator, device status indicator bar, and real-time information display bar.

It is important to mention that when the handler receives a request for data, it takes it out from the corresponding table and attributes it to the address of the information object, which is packaged and sent to the command station. After passing through the units and being evaluated, the last step is to release the data.

**Figure A5:**
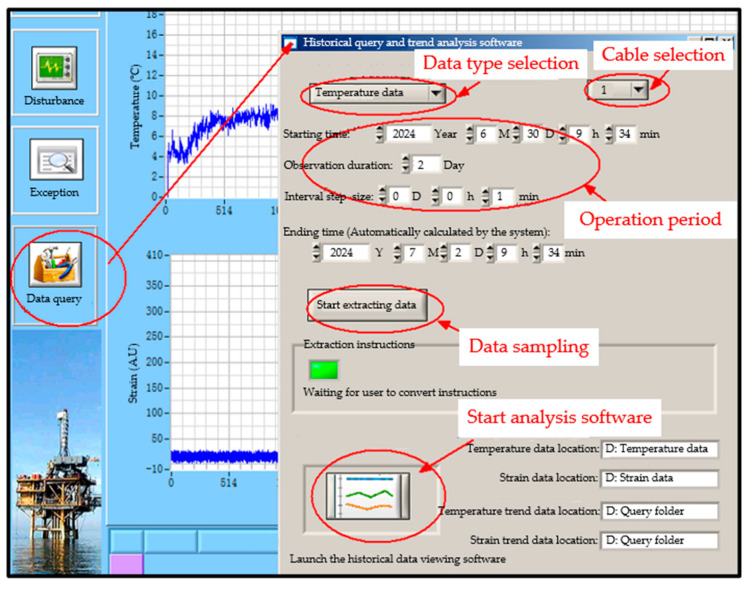
Historical data query and trend analysis interfaces.

Once the data analysis software is launched in shortcut mode, click on the “Data Query” box in the main interface. The data query provides a historical data query and trend analysis interface, which has two functions: single-day data viewing and sampling data analysis. The list of released data files for each data type can be found there, as shown in Figure A5 (See Appendix A).

View the release data files by selecting the “Data Query” box, and then choose the desired data type among the three. If the data to be released are related to cable 1, then cable 1 is selected, as shown below.

### 5.4. Performance Test and Analysis

#### 5.4.1. Electrical Energy Data Release

Usually, the temperature of the optical fiber in the submarine cable is the same as that of its insolation. Therefore, in the experiment, the optical fiber of the cable is input into a tank containing water at a temperature of 20 °C. The current is adjusted through a large generator fixed on the tank, which involves changing the temperature of the water, making it possible to have different loads of electrical energy in the cable. After applying different current loads (100A, 200A, …, 1000A) under different thermal conductivities, the temperature of the fiber before correction was obtained, as displayed in Table A8 (See Appendix A).

**Figure 7:**
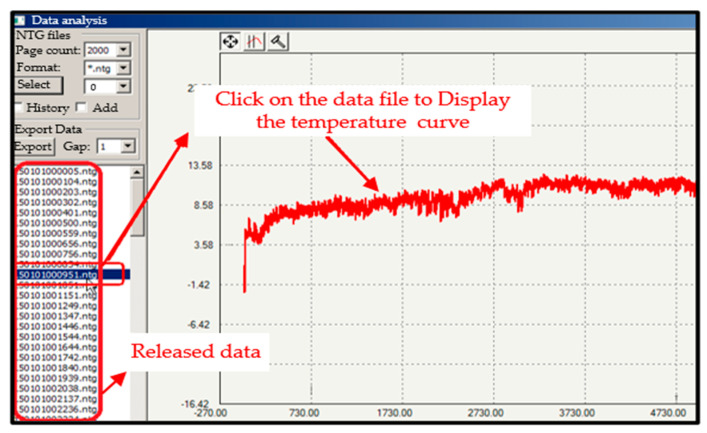
Temperature trend interface.

A total of 80 datasets (eight datasets for each load of current) were collected. After the parameter correction and assessment, the final electrical energy was achieved and sent to the command station. Of the 80 datasets sent, 79 were received with a transmission time of about 0.103 s. A list of the released data files can be found in the temperature trend interface, as shown in Figure 7. View this released data by clicking on “Data File” in the interface; the resulting data curve is displayed. Then, click on any physical point on the trend temperature curve to view the electrical energy of that period.

#### 5.4.2. Disturbance Data Release

**Figure 8:**
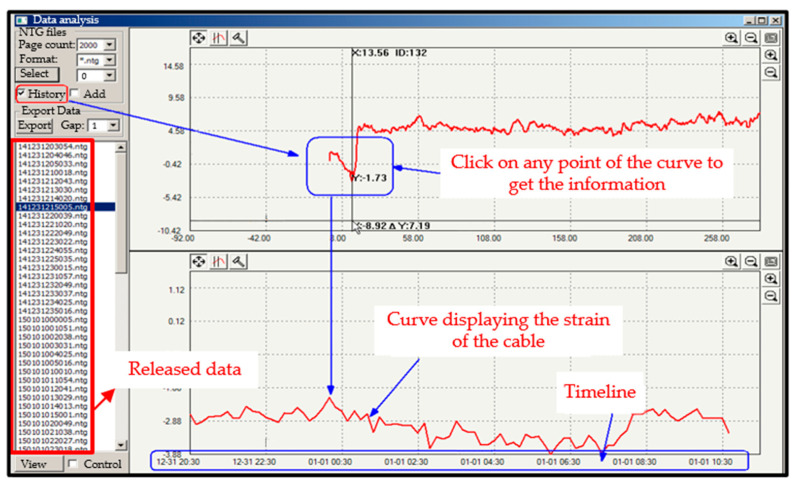
Disturbance trend interface.

Using the case of collision events with cable 3, experiments are carried out on a total of 120 datasets. Three classes of data are registered, as presented in Table A3 (See Appendix A); each class has 40 datasets. Among the 120 datasets, 117 were received by the command station with a transmission time of about 0.149 s. Of the three datasets not transmitted, two came from the second class and one from the third class. A list of the released data files can be found in the disturbance trend interface displayed in Figure 8. View this released data by clicking on “Data File”, then click on the disturbance trend curve where the physical location point of the collision appears to see the magnitude and the time of the collision event.

#### 5.4.3. Vessels Positioning Data Release

A total of 50 vessels are used in the experiments. AIS downlink signals include a Doppler frequency shift, which is up to a maximum of ±5 kHz. The system supports simultaneous access and dynamic configurability of multiple devices, as a single AIS device monitors the sea area with a limited range and low stability. The system operates in such a way that after localizing and judging vessels entering the warning zone, a short message stating “You are entering the precautionary area of the submarine cable, please, an anchorage cannot be allowed!” is sent to the vessels. The indicators of this system are listed in Table A9 (See Appendix A).

**Figure 9:**
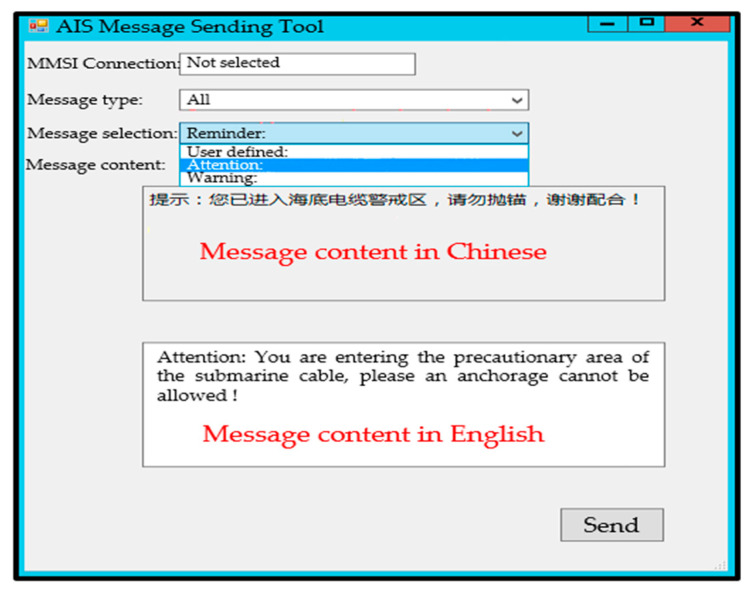
Controlled message-sending interfaces.

The 50 vessels were localized, but 49 of them received a short message sent by the command station, with a processing time of approximately 0.0108 s. Controlled messages can be sent manually or automatically to the target vessels using the MMSI number. As displayed in Figure 9, the message states “You are entering the precautionary area of the submarine cable, please, an anchorage cannot be allowed!”.

**Figure 10:**
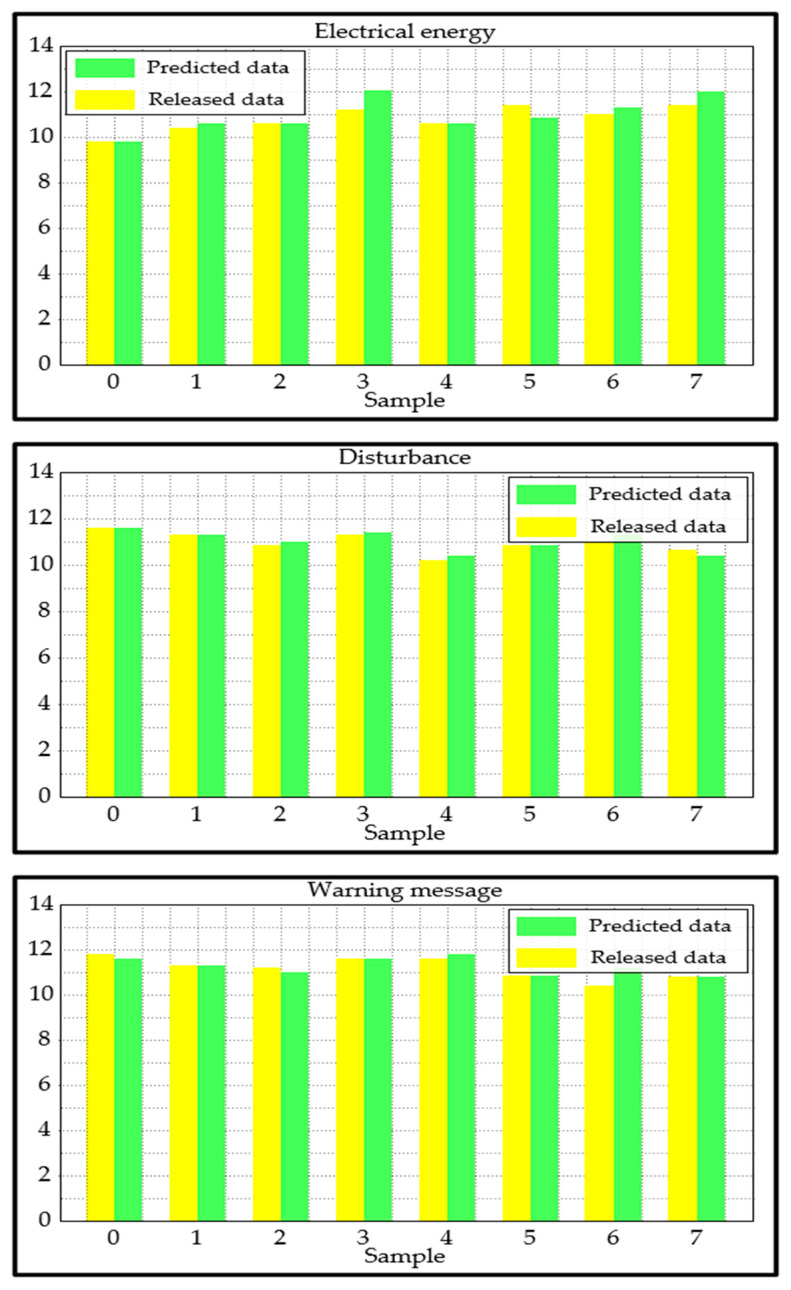
Errors in eight samples of the test dataset.

Figure 10 displays the results for the eight samples to demonstrate the performance on the test dataset that the proposed approach was able to achieve.

- We can draw the conclusion that, with very few exceptions, the three variables used in the test are predicted quite accurately.
- We can see that regardless of the sample number, there is no significant discrepancy between the three cases.
- The disturbance data are labeled with the highest relative discrepancy in the fourth sample, resulting in a label that does not perform well compared with the other two cases. However, it is evident that the proposed approach produced an acceptable performance.

As a result, we believe that using data from several sources, including electrical energy, disturbance, and vessel detection, along with a warning message, offers useful information for the online monitoring system.

Although the proposed approach achieves the best results in terms of data release, it is not always realistic. The primary constraint is that we are dealing with a time-series problem, and multiple factors need to be taken into account. In addition, the approach only takes into account the data released from a single department during a certain period. For example, data on electrical energy and disturbance of the same cable are released at the same time. In this instance, the test dataset must contain all time-series sequences associated with this procedure; they cannot be dispersed across the two datasets.

## 6. Validation of the Approach

**Figure 11:**
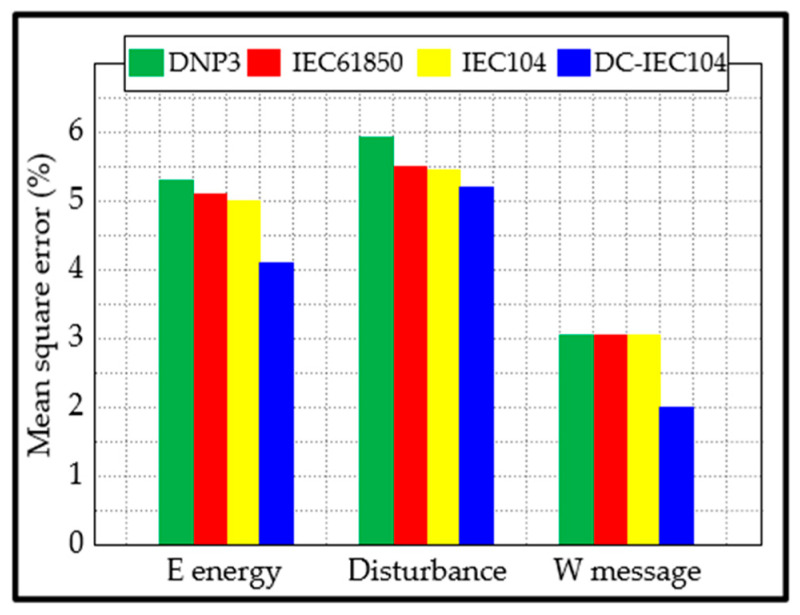
Mean squared error of the test dataset.

The same experiments are performed using the same datasets; these data are released using three commonly used protocols: DNP3, IEC61850, and IEC104. The aim is to compare the results obtained by these approaches with those obtained using the proposed approach. The mean square error (MSE) is applied to evaluate the performance of all these approaches. This is defined in Equation (16).

(16) $$MSE=\frac{1}{n}\sum_{i=1}^{n}{\left(Y_{i}-{\hat{Y}}_{i}\right)}^{2}$$

where Y and $\hat{Y}$ are the real data and the predicted data, of each sample i, respectively; n is the total samples. After computation, the results of the mean squared error for each approach are displayed in Table 10 and Figure 11. Table 11 shows the time spent on data releases.

A closer examination of the results reveals the following:

- The average MSE of our approach is 3.78%, while those of DNP3, IEC61850, and IEC104 are 4.74%, 4.53%, and 4.50%, respectively.
- The data release time for disturbance using DNP3 was slightly better than that of our approach. This may be because DNP3 performs very well in transmitting a larger amount of data (but few data packets) over longer distances, compared with our approach, which transmits a high number of small data packets.
- With the exception of the data release time for disturbance, our approach has a better consumption time.

In addition, advantages that are not found in other approaches can be mentioned as follows.

- The proposed approach ensures that data from each monitoring device only enters the appropriate subunit.
- The proposed approach directs and observes how commands are executed in the actuator.
- The proposed approach guarantees that the commands produce effects that ultimately correct the anomalies measured by the sensors.
- It can independently make corrections based on the choices made by the servo application layer.
- The OC returns data to complete the sending of an APDU response upon receiving the data (release request) and returning the equivalent data (release response). If APDU chaining is employed, the final APDU response of a chain is obtained.
- A value set to ‘FFFF’ indicates that the data management unit can send almost any quantity of data. The OC may suggest a suitable value for this scenario.

### 6.1. Evaluation Protocol

We assess the precision, recall, and F-score of the data release to determine the quality of each approach. We consider $N_{i}$ being the data collected by the command station (point A), and which must be released to the controlled station 1 (point B); $M_{i}$ is the released data at point B. We aim to identify a match such that $N_{i}$ and $M_{i}$ are equal. At present, we can determine true positive (TP), false positive (FP), and false negative (FN) in accordance with the matching of $N_{i}$ and $M_{i}$. A TP is the predicted positive dataset, an FP is a false alarm of the dataset, and a FN is a failure to detect a positive dataset. The formulas for determining the precision, recall, and F-score are as follows [49,50]:

(17) $$P=\frac{TP}{TP+FP},\;R=\frac{TP}{TP+FN},\;F=\frac{2PR}{P+R}$$

Several data samples were collected and released for each device/sensor, as indicated earlier in this section. As a result, we obtain the precision, recall, and F-score for each method. The performance evaluations are presented in Table 12.

The results in Table 12 show that the proposed approach yields the best results in terms of precision, recall, F-score, and processing time; this confirms its effectiveness.

### 6.2. Performance Comparison

Three approaches, including a reasonable correlation analysis method for data release (AP.1) [51], a blockchain-based mechanism for timed data release and timed transactions (AP.2) [52], and a novel outsourcing differential privacy data release scheme (AP.3) [53], are used for data release. Among them, AP.1 combines feature-matching algorithms with information entropy-based feature importance. This approach provides a data release solution to reduce data dimensionality and improve the training efficiency of machine learning by combining the maximum information coefficient with differential privacy. AP.2 uses a basic protocol that consists of two critical ingredients: reputation-aware peer recruitment and verifiable enforcement protocols. This makes the design probabilistically attack-resilient to post-facto attacks. AP.3 uses a new differential outsourcing system for privacy data release, which allows data providers to outsource their datasets to a cloud service provider at a low communication cost.

A total of 50 datasets for each sensor were collected during each season of the 2024 calendar year (spring, summer, autumn, and winter) for the transmission of data from the command station (point A) to controlled station 1 (point B). This data collection and transmission have been continuously running without interruption of the Internet connection. Using the same information provided in Section 6.1 on TP, FP, and FN, the results obtained by applying the four approaches are presented in Table 13.

We can observe that, with the exception of the fact that we have not yet implemented security, scalability, and maintenance in our system, its average results are better than those of other approaches. Our system has the lowest error rate, the lowest data transmission time, and the highest data transmission accuracy. However, we have realized that, during the summer, these characteristics decrease. We believe that this decrease is due to the fact that the devices/sensors are overheating. Therefore, the security, scalability, and maintenance of these devices/sensors play a major role in continuous, sustainable, and reliable operation.

## 7. Discussion

### 7.1. Comparative Discussion

Based on the papers we have read, we found that sensing, monitoring, and data release are the most common applications of IoT in instrumentation and measurement. Approximately 67% of these papers concentrate on using the IoT to expand traditional measuring systems to accomplish wide-area continuous monitoring. However, the IoT also includes actuators in addition to sensing. None of these papers provides extended and modified solutions that could potentially enhance IoT systems. The measurement of IoT network latencies is the subject of these papers. These papers aim to pinpoint the IoT’s data limitations with regard to coverage and data transfer speed. Regrettably, many mistakenly believe that the IoT is only applicable to homogeneous networks, which is untrue. The function of an IoT gateway involves connecting devices that utilize diverse network technologies and anticipating future protocol expansions. Therefore, our system uses the DC-IEC104 protocol and file transfers to release any kind of data (complicated/uncomplicated, large/small volumes), which effectively improves system compatibility.

Currently, it is difficult to statistically compare our system with other systems because our system is more developed for data release, while other systems are more developed for security, scalability, and so on. The final statistical validation of the reliability of our system and the comparison of our entire system with other systems can be performed effectively once we have completed addressing the issues mentioned in Section 6.2.

### 7.2. Discussion on Weaknesses and Challenges

To collect, process, and release the data, we have introduced a three-layer IoT architecture. The data media layer is the first layer of the structure and contains all the monitoring devices/sensors of the system. It is responsible for acquiring and transmitting data to the OC in the control layer. The control layer is responsible for receiving and transmitting data to the servo application layer. These data will be processed and released between the platforms.

The proposed method is applied to a real-world scenario presented in Section 5. To date, no problems in the process of data collection, processing, and transmission have been encountered; that is, the results recorded so far are still constant. This proves the effectiveness and reliability of the proposed approach. However, we still need to address the information leakage hazards of our system.

- Online invasions may result from inadequate encryption and authentication. Monitoring devices/sensors may be susceptible to hacking due to the lack of adequate security mechanisms. Infected IoT nodes can jeopardize the integrity of the entire smart structure. Cybercriminals can invade IoT networks, rendering smart structures unusable. Therefore, to avoid such malicious attacks, adequate encryption and authentication must be performed.
- Over time, we know that the number of devices could increase, which will also increase the connection; the need for data transmission and storage will increase accordingly. New communication protocols can be used for this purpose; moreover, edge computing and cloud infrastructure must adapt to increasing data loads.
- Patching must be performed frequently to prevent security lapses. Long-term operation, especially in isolated or maritime environments, depends on effective energy management. Therefore, the use of artificial intelligence-based analysis will be significant for optimizing maintenance cycles and predicting malfunctions.
- We have noticed that during summer, the monitoring devices/sensors heat up so much that they are subject to crashing. Therefore, we designed a microcontroller that can reset the service while saving data when it crashes [54]. However, it is important to always regulate the temperature of the control room where the monitoring devices/sensors are placed so that they are free from overheating.

## 8. Conclusions and Future Work

An increasing number of marine operations and offshore fields are automating their processes to improve security and workloads. This automation is carried out using sensors/actuators to collect data that are analyzed, evaluated, and released, allowing managers to receive commands and take timely intervention actions to avoid equipment loss and economic crisis.

In this paper, a novel three-layer IoT architecture is proposed that uses the DC-IEC104 protocol to carry out a continuous execution of data release. It provides the necessary mechanisms for collecting, analyzing, evaluating, and releasing data. The proposed approach emphasizes the DC of the system and the key technology for massive data transmission. Experiments were carried out, and the results obtained show that the proposed approach achieved a lower average mean square error of 3.78 and a lower average time consumption of 1.083 s compared to those obtained with other approaches. In addition, the results obtained for the precision, recall, and F-score prove that the proposed approach leads to better performance. Almost all data input in the data release protocol unit were transmitted to the command station without any problems.

In future work, we plan to achieve the following three objectives:

- We need to add an extra layer since the Servo Application Layer is saturated with multiple modules and units. This will allow the system to avoid clutter, improve data processing, and reduce the release time.
- We will consider including new types of information sources, such as images of submarine cables captured at sea. This will allow us to obtain graphical information on all the cables so that, in addition to the technical information code, the graphic image can also be released on the appropriate platform.
- The application of the measure unit through its commands produces effects that can correct the abnormality measured by the sensors. In addition, the OC can take autonomous corrective measures. However, these measures are sometimes not the most effective. Thus, we must continue to work on finding a solution to this problem.
- The points mentioned in the Discussion section will be considered to improve the operation of our system in terms of security, scalability, and maintenance.

After completing our future work, we believe that the new AI-integrated IoT system will drive the adoption of autonomous vessels. It will monitor the state of the oceans, pollution levels, and marine biodiversity. Moreover, it will strengthen the defenses against cyber threats to maritime operations.

## Figures and Tables

**Table 1:** Simulation environment.

Hardware	Memory	16 GB RAM
	CPU	Intel(R) Xeon^®^ E5-2620 v3 @ 2.40 GHz 2.40 GHz (Anxin Information Technology Co., Ltd., Shanghai, China)
Software	Library	API
	Programming Language	Matlab R2016a (8.3.0.532) and LabWindows/CVI
	Operating System	Window 10

**Table 2:** Types and amounts of data to be transmitted.

Amount of Data	Amount of Data	
	Simple Data	Complex Data
Small amount of data	Electric energy, equipment status	Alarm, AIS
Big amount of data	Temperature, strain, disturbance	-

**Table 3:** Allocation of information object addresses.

Information Object Name	Corresponding Address (Hex)	Amount of Information	Table	Field	Data Type
Electrical energy	000001H~005000H	20,480	Current	Electrical energy	Floating-point
Cladding monitoring	005001H~00A000H	20,480	Protective Layer	Cladding monitoring	Floating-point
Device status	00A001H~00A200H	512	State	Device status	Integer

Hex = Hexadecimal.

**Table 4:** Mapping the relationship between the file name and the information object.

File Section Name	Information Object
1	Temperature
2	Strain
3	Disturbance
4	Alarm
5	AIS
-	-

**Table 5:** Type identification of the file transfer.

Message Type (Decimal)	Message Semantics	Encode
120	The file is ready	F_FR_NA_1
121	The section is ready	F_SR_NA_1
122	Summon directory, select file, summon file, summon section	F_FC_NA_1
123	Final section, last paragraph	F_LS_NA_1
124	Confirm the file, confirm the section	F_AF_NA_1
125	Segment	F_SG_NA_1
126	Directory	F_DR_NA_1
127	Retain	-

**Table 6:** Directory asdu definition of file transfer (type identification: f_dr_ta_1).

ASDU Definition	Number of Bytes
Type Identification (TYP)	1
Variable Structure Qualifier (VSQ)	1
Reason for transmission (COT)	2
ASDU Public Address (CA)	2
Information Object Address (IOA)	3
File name or subdirectory name (NOF)	2
File length (LOF)	3
Status of the file (SOF)	1
File creation time (CP56Time2a)	7

**Table 7:** Segment asdu definition of file transfer (type identification: f_sg_na_1).

ASDU Definition	Number of Bytes
Type Identification (TYP)	1
Variable Structure Qualifier (VSQ)	1
Reason for transmission (COT)	2
ASDU Public Address (CA)	2
Information Object Address (IOA)	3
File name (NOF)	2
Section Name (NOS)	1
Length of segment (LOS)	1
Segment	n

**Table 8:** AIS data type.

AIS Data Information	Data Type	Number of Bytes
MMSI Identifier	String	9
Device type	String	1
Name of the ship	String	10
Longitude	Floating-point	4
Latitude	Floating-point	4
Name of the nearest submarine cable	String	10
Nearest submarine cable distance	Floating-point	4
Speed	Floating-point	4
Course	Floating-point	4

**Table 9:** Alarm data type.

AIS Data Information	Data Type	Number of Bytes
Alarm number	Integer	4
Alarm time	String	20
Submarine cable number	Integer	4
Cable details	String	10
Start position	Floating-point	4
End position	Floating-point	4
Alarm code	Integer	4
Alarm details	String	20
Processing status	String	10

**Table 10:** Mean error for the data release.

	Approaches			
Mean Square Error (%)	Using DNP3	Using IEC61850	Using IEC 104	Using DC-IEC 104
Electrical energy	5.23	5.02	5.00	4.11
Disturbance	5.93	5.51	5.49	5.27
Ships positioning	3.06	3.06	3.06	1.99

**Table 11:** Time consumption for data release.

	Approaches			
Time Consumption (s)	Using DNP3	Using IEC61850	Using IEC 104	Using DC-IEC 104
Electrical energy	1.771	1.104	1.108	1.103
Disturbance	1.106	1.245	1.149	1.149
Ships positioning	1.315	1.123	1.113	1.110

**Table 12:** Evaluation of the different methods.

	Approaches			
Characteristics	Using DNP3	Using IEC61850	Using IEC 104	Using DC-IEC 104
Number of data released	245	244	243	245
Number of false positive	2	0	1	0
Number of unreleased data	5	6	7	5
False data release rate	0.47	0.045	0.045	0.037
Average precision	0.879	0.898	0.883	0.902
Average recall	0.888	0.909	0.913	0.987
Average F-score	0.923	0.941	0.921	0.951
Average processing time	1.398 s	1.124 s	1.090 s	1.083 s

**Table 13:** Comparison of the results of the different approaches.

Characteristics		Approaches			
		AP.1	AP.2	AP.3	DC-IEC104
Security		√	√	√	×
Scalability		√	√	√	×
Maintenance		√	√	√	×
Spring (10 °C~25 °C)	Number of data	50	50	50	50
	Precision	90.17%	89.94%	90.11%	91.29%
	Error rate	2.52%	4.76%	4.34%	2.21%
	Processing time	1.371 s	0.988 s	2.336 s	1.083 s
	Remark	Regular	Regular	Regular	Regular
Summer (26 °C~31 °C)	Number of data	50	50	50	50
	Precision	90.17%	89.94%	90.11%	88.73%
	Error rate	2.52%	4.76%	4.34%	4.33%
	Processing time	1.371 s	0.988 s	2.336 s	1.174 s
	Remark	Regular	Regular	Regular	Regular
Autumn (16 °C~24 °C)	Number of data	50	50	50	50
	Precision	90.17%	89.94%	90.11%	91.29%
	Error rate	2.52%	4.76%	4.34%	2.21%
	Processing time	1.371 s	0.988 s	2.336 s	1.083 s
	Remark	Regular	Regular	Regular	Regular
Winter (−4 °C~11 °C)	Number of data	50	50	50	50
	Precision	90.17%	89.94%	90.11%	91.29%
	Error rate	2.52%	4.76%	4.34%	2.21%
	Processing time	1.371 s	0.988 s	2.336 s	1.083 s
	Remark	Regular	Regular	Regular	Regular

°C = Degree Celsius.

## Data Availability

The data used in this research work are unavailable due to ethical restrictions.

## Appendix A

**Table A1:** Single-energy data transmission configuration information.

Configuration Information	Parameter	Interpretation
TypeID	9	The type identifier is a normalized telemetry value
InformationObjectAddress	1	The information object address is 1
Quantity	1	The number of messages is 1
TransmitionCause	20	The reason for the transmission is a response station call
TransmissionTime	60	The transmission frequency is 60
CommonAddress	1	The public address is 1

**Table A2:** The command station receives the normalized telemetry message with type ID = 9.

Message	Message Explanation
68	Initiate
10	Length
0600	Send sequence number
0200	Receive the serial number
09	Type ID 9, telemetry with quality description
01	Variable structure qualifier, a telemetry data
1400	Transmission reason 14, response to total call
0100	Public address
01000	Information object address, starting from 0x0001 telemetry number 0
a110	The telemetry value is 10a1
00	Quality descriptor

**Table A3:** Data sheet.

Class of Data	Forces	Frequencies
First class of data	0.00 N	0 to 10,000 kHz
	5.00 N	…
	…	…
	…	…
	16.00 N	0 to 10,000 kHz
Second class of data	17.00 N	0 to 10,000 kHz
	20.00 N	…
	…	…
	…	…
	58.00 N	0 to 10,000 kHz
Third class of data	59.00 N	0 to 10,000 kHz
	70.00 N	…
	…	…
	…	…
	100.00 N	0 to 10,000 kHz

kHz = kilohertz, N = Newton.

**Table A4:** Disturbance data file transmission configuration information.

Configuration Information	Parameter	Interpretation
TypeID	125	The type is identified as file segment transfer
TransmitionCause	13	The reason for the transfer is file transfer
CommonAddress	1	The public address is 1
Information ObjectAddress	0	The information object address is 0
FileName	1	The file name is 1, temperature file
FileLength	150	The file length is 750 KB
SectionName	1	The section name is 1
SectionLength	7680	The length of the section is 7680 B
SegmentLength	120	The segment length is 120 B
TransmissionTime	60	The transmission frequency is 60

**Table A5:** The command station receives the segment message with type ID = 7d.

Message	Message Explanation
68	Initiate
12	Length
0600	Send sequence number
0200	Receive the serial number
7d	The type identifies 7d, segment
01	Variable structure qualifier
0d00	Transfer reason, file transfer
0100	Public address
000000	The address of the information object
0110	File name, temperature data file
01	Section name
78	Segment length
01	Segment

**Table A6:** Dual station system in the configuration file.

Joint	Key 1	Key 2
Total system configuration	Number of clients	Number of AIS devices
AIS equipment No. 1	Device serial port number	AIS MMSI number
AIS equipment No. 2	Device serial port number	AIS MMSI number
Client 1	IP address	IP port number
Client 2	IP address	IP port number

**Table A7:** Intelligent message type.

Message Type	Message Type Number	Remarks
Broadcast binary message	Message 8	It can send both Chinese and English
Broadcast security message	Message 14	It can only send English
Addressing binary messages	Message 6	It can send both Chinese and English
Addressing security messages	Message 12	It can only send Chinese

**Table A8:** Actual data sheet.

Input Current (A)	100	200	300	400	500	600	700	800	900	1000
Original fiber temperature (°C)	16.602	16.980	17.609	18.491	19.625	21.011	22.648	24.609	26.679	29.073

**Table A9:** Technical indicators.

Technical Pointers	Parameter Value
Number of ships handled by the server per unit of time	50 ships
Decoding accuracy of the unit time of the acquisition module	98%
Acquisition module receiving/time unit the number of ships	49 ships
The server handles the correct rate of the ship per unit of time	98%
AIS package analysis shows a delay	10 ms
Short message system response delay	10 ms
Historical data query response delay	10 ms
Number of ships per unit time	38 ships
